# A Novel Framework to Predict Breast Cancer Prognosis Using Immune-Associated LncRNAs

**DOI:** 10.3389/fgene.2020.634195

**Published:** 2021-01-21

**Authors:** Zhijian Huang, Chen Xiao, Fushou Zhang, Zhifeng Zhou, Liang Yu, Changsheng Ye, Weiwei Huang, Nani Li

**Affiliations:** ^1^Department of Breast Surgical Oncology, Fujian Medical University Cancer Hospital, Fujian Cancer Hospital, Fuzhou, China; ^2^Breast Center, Nanfang Hospital, Southern Medical University, Guangzhou, China; ^3^Department of Gastroenterology, Fuzhou Second Hospital Affiliated to Xiamen University, Fuzhou, China; ^4^Department of General Surgery, The Hospital of Changle District, Fuzhou, China; ^5^Laboratory of Immuno-Oncology, Fujian Medical University Cancer Hospital, Fujian Cancer Hospital, Fuzhou, China; ^6^Department of Thyroid and Breast Surgery, The First Affiliated Hospital of Sun Yat-sen University, Guangzhou, China; ^7^Department of Medical Oncology, Fujian Medical University Cancer Hospital, Fujian Cancer Hospital, Fuzhou, China

**Keywords:** TCGA, GEO, immune, breast cancer, risk score, framework, prognosis

## Abstract

**Background:** Breast cancer (BC) is one of the most frequently diagnosed malignancies among females. As a huge heterogeneity of malignant tumor, it is important to seek reliable molecular biomarkers to carry out the stratification for patients with BC. We surveyed immune- associated lncRNAs that may be used as potential therapeutic targets in BC.

**Methods:** LncRNA expression data and clinical information of BC patients were downloaded from the TCGA database for a comprehensive analysis of candidate genes. A model consisting of immune-related lncRNAs enriched in BC cancerous tissues was established using the univariate Cox regression analysis and the iterative Lasso Cox regression analysis. The prognostic performance of this model was validated in two independent cohorts (GSE21653 and BC-KR), and compared with known prognostic biomarkers. A nomogram that integrated the immune-related lncRNA signature and clinicopathological factors was constructed to accurately assess the prognostic value of this signature. The correlation between the signature and immune cell infiltration in BC was also analyzed.

**Results:** The Kaplan-Meier analysis showed that the OS of Patients in the low-risk group had significantly better survival than those in the high-risk group, Clinical subgroup analysis showed that the predictive ability was independent of clinicopathological factors. Univariate/multivariate Cox regression analysis showed immune lncRNA signature is an important prognostic factor and an independent prognostic marker. In addition, GSEA and GSVA analysis as well as comprehensive analysis of immune cells showed that the signature was significantly correlated with the infiltration of immune cells.

**Conclusion:** We successfully constructed an immune-associated lncRNA signature that can accurately predict BC prognosis.

## Introduction

Globally, Cancer Statistics 2018 estimates that more than 2.1 million females are diagnosed with breast cancer (BC) every year and about 62,000 deaths occur, making it the most common female malignancy and the leading cause of cancer mortality in women. Developing countries account for nearly 60% of BC mortality (Bray et al., 2018). Although the progress of early screening and recent advances in anti-cancer strategies offer improvements in the main outcomes of BC patients (Kim et al., 2018; Siegel et al., 2020), the recurrence rate of BC remains high (Li et al., 2019). Several studies have identified a large amount of poor-prognosis related biomarkers for BC, including age, tumor size, histological grade, lymphatic vessel invasion (LVI), number of metastatic lymph nodes, hormone receptor status, c-erbB2 status, and positive margin status. However, because of the complexity of BC incidence and the heterogeneity of tumors, pre-existing prognostic markers are underpowered to increase the prediction efficiency. The emerging technique, an optimal model consisting of relevant lncRNAs, will renew hope for pronounced improvements in predicting the BC prognosis.

LncRNAs, located in the nucleus or cytoplasm, are a group of RNA molecules of greater than 200 nt in length. The majority of them have no protein-coding function, and a few can encode a limited number of polypeptides. LncRNAs are engaged in precise regulations at pre-transcription and transcription levels and a broad spectrum of biological processes, such as tumor invasion, metastasis, apoptosis, and drug resistance. Therefore, lncRNA abnormalities in the peripheral blood of BC patients may promote BC formation and progression, promoting early diagnosis and timely treatment for patients. Many studies have confirmed the regulatory effects of lncRNAs on malignancy behaviors in tumorigenesis (Mercer et al., 2009; Fang and Fullwood, 2016; Bin et al., 2018), including proliferation, adhesion, migration, and apoptosis of tumor cells (Wang et al., 2020). HOXA11-AS and MALAT1 are identified as the lncRNAs related to cell cycle progression, which can be used as prognostic biomarkers for the survival of glioma patients (Wang et al., 2016).

Immune-related lncRNAs refer to those with various functions in regulating gene expressions and critical roles (e.g., the control of the differentiation and function of immune cell types, dendritic cell activity, T cell ratio, and metabolism) in innate or adaptive immune responses (Denaro et al., 2019). Hu et al. (2013) demonstrated that a lincRNA Ccr2-5′AS was an important part of the regulatory circuit in gene expression specific to the TH2 subset of helper T cells. Yang et al. (2020) found that LNC TANCR play important roles in γδ T cell activation. Huang et al. (2018) noticed that lncRNA NKILA, regulated T cell sensitivity to Activation-induced cell death (AICD) by inhibiting NF-κB activity. However, details of immune-related lncRNAs related to BC are rarely reported. In this study, an immune-related lncRNA signature was constructed and verified in an independent set and its association with immune cell infiltration was analyzed to evaluate the clinical predictive value of the signature.

## Materials and Methods

### Data Download, Pre-processing and Screening of Prognostic IRGs

To identify BC-related lncRNAs, lncRNA profile, and clinical data from the TCGA training set were downloaded from the UCSC Xena database^1^; To verify the multi-lncRNA signature, gene profile and clinical information of the Breast Cancer-KR (BC-KR) data set were downloaded from ICGC^2^; GEOquery package with R language (Davis and Meltzer, 2007) was used to download and analyze the reliable BC expression data set GSE21653 from the GEO database, the source of species was human, and the platform was based on the GPL570 ([HG-U133_Plus_2] Affymetrix Human Genome U133 Plus 2.0 Array), including 266 BC samples included in this study. The BC-KR data set and the GSE21653 data set were used as verification sets. Firstly, the gene expression data and the corresponding clinical data of BC patients were pre-processed; the immune response genes (IRGs) were downloaded and sorted out from ImmPort database^3^. The prognostic immune-related lncRNAs were screened out from the TCGA data set using the univariate Cox regression method, and the statistical standard of *p* < 0.05 was used. We performed GO and KEGG enrichment analysis to extract prognostic IRGs (Supplementary Figure 1).

### Screening, Co-expression Analysis and GO Enrichment Analysis of Immune-Related LncRNAs

The gene list of expression matrix was annotated and classified by mRNA and lncRNA, the mRNA expression matrix and lncRNA expression matrix were distinguished, and Pearson coefficient between lncRNA gene expression and prognostic IRGs was calculated. Pearson coefficient <−0.4 and *P* < 0.01 were set as the identification criteria of lncRNAs related to immune. In order to explore the possible function of lncRNAs related to immune in the occurrence and development of BC, co-expression analysis was performed on mRNA expression matrix and immune-related lncRNAs expression matrix. GO enrichment analysis was performed to annotate the immune functions of target immune-related lncRNAs using the ClusterProfiler package (Yu et al., 2012). Adj *p* value < 0.05 was considered statistically significant.

### Construction and Evaluation of a Prognosis Model

A least absolute shrinkage and selection operator (Lasso) Cox regression model was used to identify optimal lncRNA candidates and construct the immune-related lncRNA signature. The process of cross-verification is to select samples randomly. The optimal value of λ was identified to train the model. Optimal lncRNA candidates were included during the training and stopped when the area under the curve (AUC) of ROC reached the peak. Therefore, the optimal multi-lncRNA signature containing the least number of prognostic immune-related lncRNAs was refined (Tibshirani, 1997). Subsequently, a univariate Cox regression analysis was implemented to identify and confirm prognostic immune-related lncRNAs in the signature, with *p* < 0.05 as the standard. An iterative Lasso Cox regression analysis was performed to construct the prognostic immune-related lncRNA signature using the glmnet package (Friedman et al., 2010). The specific method was to count the consensus genes whose gene frequency is more than 100 after 1000 Lasso Cox regression. Then, a multivariate Cox regression analysis was conducted to assess the prognostic capacity of the refined prognostic gene signature, using the expression values of genes as covariates. Subsequently, a prognostic risk signature was conducted for each patient from the TCGA training cohort according to the refined prognostic gene-expression signature. Using the median risk score as the threshold, these patients were divided into the high- and low-risk groups and the survival differences between the high- and low-risk groups were analyzed using the R software.

### Comparison of the Prognostic Multi-LncRNA Signature With Known Biomarkers and Validation of the Signature

To explore the prognostic capability and prediction efficiency of our signature for BC, a comparative survival analysis was used to compare the prognostic capability between the signature and known prognostic biomarkers, and the ROC curve was used to analyze the sensitivity and specificity of the prognostic risk signature. Based on the prognostic multi-lncRNA signature in the training set, its prognostic performances were verified in two independent validation sets (BC-KR and GSE21653 validation sets).

### Subgroup Survival Analysis, the Prognostic Value of the Signature, and Construction of Nomograms

The prognostic value of any biomarker can be evaluated by whether they are independent of the existence of clinicopathological prognostic factors. In this study, to evaluate the independence and applicability of the validated signature, the BC patients in the TCGA cohort were reassigned according to different clinicopathological characteristics. Kaplan-Meier survival analysis was performed to compare the prognostic capability between subgroups. Decision curve analysis was used to evaluate the net benefit and clinical value of the signature. Univariate Cox regression and multivariate Cox regression were used to evaluate the associations between clinical features and the risk score values, which were illustrated as nomograms.

### Phenotype Differences Between the High- and Low-Risk Groups Using GSEA and GSVA Analysis

Differences in functional phenotypes between the high- and low-risk groups were analyzed using the gene set enrichment analysis (GSEA) and GSVA analysis. We selected “c2.all.v7.1.symbols.gmt,” “c5.all.v7.1.symbols.gmt,” and “h.all.v7.1 symbols.gmt” as the reference gene sets. The differences in activated pathways between the high- and low-risk groups were identified using GSEA v4.0.3 software, and permutations of the gene sets were performed 1000 times for each analysis. A nominal *p* < 0.05 and a false discovery rate (FDR) < 0.05 were considered as statistical significance. We selected “h.all.v7.1 symbols.gmt” as the reference gene set. The differences in activated pathways were also analyzed using the ClusterProfiler package and GSVA package (Hänzelmann et al., 2013). A *p* value < 0.05 was considered statistically different.

### Correlation Between the Prognostic Signature and Immune Cell Infiltration

To explore the association between the lncRNA signature and immune cell infiltration in BC, gene expression matrix data was uploaded to CIBERSORT (Newman et al., 2015) for cell type-specific gene expression purification. Infiltrating immune cells were filtered for analysis, and the abundances of infiltrating immune cells between the high- and low-risk groups were compared with CIBERSORT *p* < 0.05 for the eligible samples. Survival-related infiltrating immune cells were identified using Kaplan-Meier survival analysis. Finally, the correlation analysis was performed for the obtained prognostic biomarkers and immune cell infiltration. The results were visualized by using ggplot2 package (Ginestet, 2011).

## Results

### Screening of Immune-Related LncRNAs for the Prognosis of BC Patients

**FIGURE 1:**
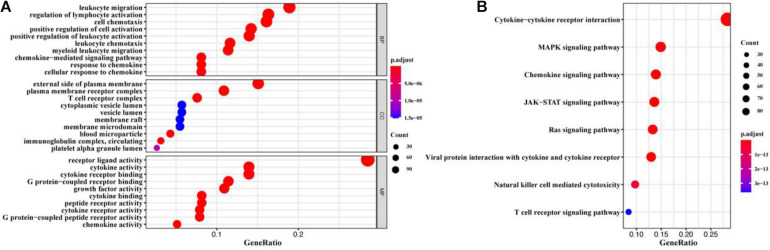
GO and KEGG enrichment analysis of prognostic IRGs. **(A)** GO enrichment result. **(B)** KEGG enrichment result. The *x*-axis represented the ratio of the number of genes enriched in a single GO (or KEGG) term to the total number of genes changed in all GO (or KEGG) terms, and the *y*-axis represents GO (or KEGG) terms. The color of the circle indicated the adjusted *p* value, and the size represented the number of genes enriched in the term or the pathway.

A total of 1900 IRGs were retrieved from the ImmPort database. Univariate Cox regression analysis showed that 516 IRGs (Supplementary Table 1) were associated with the prognosis of BC patients. We further identified immune functions of the prognostic IRGs via the gene GO and KEGG enrichment. We found that the 516 prognostic IRGs were significantly enriched in GO terms related to leukocyte migration, regulation of lymphocyte activation, cell chemotaxis, external side of plasma membrane, plasma membrane receptor complex, T cell receptor complex, and receptor-ligand activity, and in KEGG pathways related to cytokine-cytokine receptor interaction, MAPK signaling pathway, chemokine signaling pathway, and JAK-STAT signaling pathway (Figure 1 and Supplementary Table 2).

### Co-expression Analysis and GO Enrichment Analysis of Immune-Related LncRNAs

**FIGURE 2:**
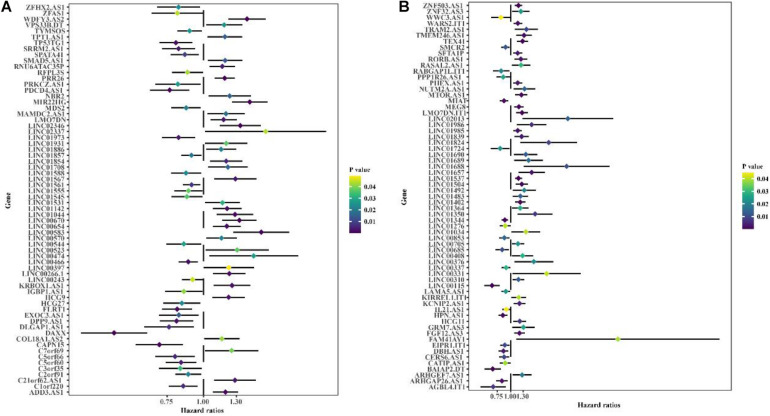
Univariate Cox regression is used to identify the forest graph of 166 immune-associated lncRNAs related to prognosis. **(A)** Part A of 166 immune-associated lncRNAs. **(B)** Part B of 166 immune-associated lncRNAs. The yellower the color is, the higher the *p* value; the bluer the color is, the smaller the *p* value.

**FIGURE 3:**
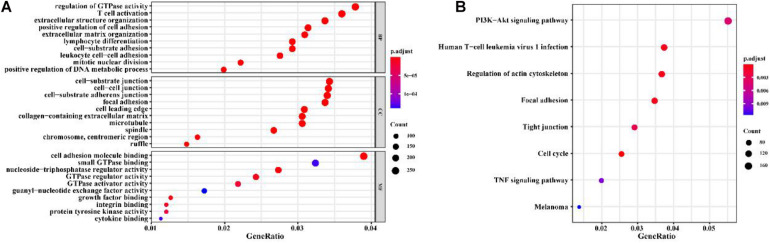
GO and KEGG enrichment analysis for the immune-related lncRNAs co-expressed with prognostic mRNAs in BC. **(A)** GO enrichment result. **(B)** KEGG enrichment result. The *x*-axis represents the ratio of the number of genes enriched in a single GO (or KEGG) term to the total number of genes changed in all GO (or KEGG) terms, and the *y*-axis represents the GO (or KEGG) terms. The color of the circle indicates the adjusted *p* value, and the size represents the number of genes enriched in the term or the pathway.

Co-expression analysis showed 948 immune-related lncRNAs linked to immune responses in BC (Supplementary Table 3). To explore the prognostic capacity of the immune-related lncRNAs, univariate Cox regression analysis was implemented and identified that 166 immune-related lncRNAs (Supplementary Table 4) were related to the maximum prognostic value (*P* < 0.05, Figure 2; *P* < 0.05). To understand the immune function of the 166 genes, we performed functional enrichment analysis for these genes. GO enrichment analysis revealed that these genes were mainly enriched in GO terms related to the regulation of GTPase activity, T cell activation, extractable structure organization, cell-substitute junction, cell adhesion, and molecular binding (Figure 3 and Supplementary Table 5). These results suggest that immune-related lncRNAs may participate in the occurrence and development of BC by regulating immune responses.

### Construction and Evaluation of the Prognostic Model

**FIGURE 4:**
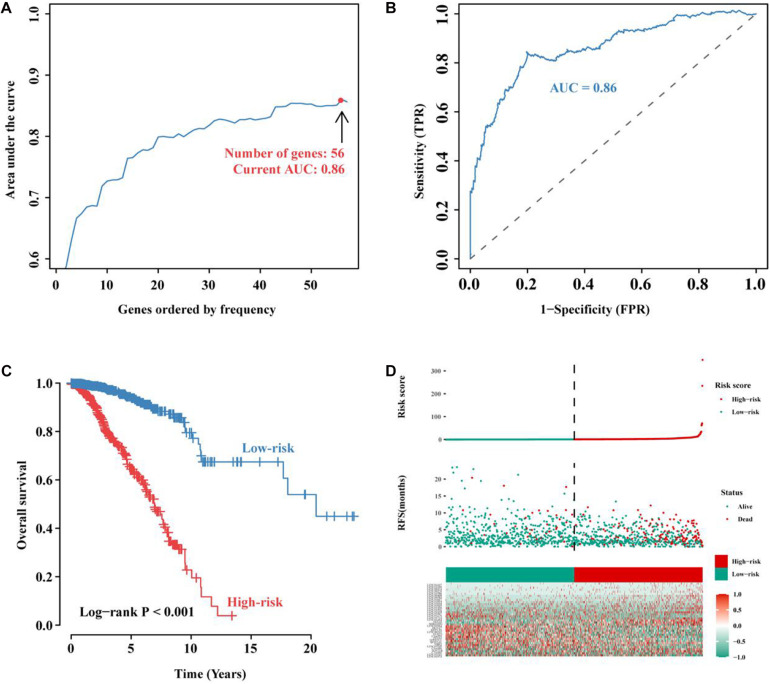
Construction and evaluation of the immune-related 56-lncRNA signature. **(A)** The immune-related lncRNA signature constructed by the iterative Lasso Cox regression analysis. **(B)** ROC curve used to evaluate the predictive efficiency of the signature. **(C)** Kaplan-Meier curve for the OS of BC patients in the high- and low-risk groups. **(D)** Risk-score distribution. In the *x*-axis, red represents higher expression levels while green represents lower expression levels. Risk scores for BC patients in the high- (red, the *y*-axis) and low-risk groups (green, the *y*-axis) were listed in ascending order.

To further improve the prediction efficiency of the signature, we performed iterative Lasso Cox regression analysis and built an optimal signature containing 56 immune-related lncRNAs (Figure 4A). To verify its predictive efficiency, we performed ROC analysis and showed that the immune-related 56-lncRNA signature had a good prognostic performance in BC (AUC = 0.86, Figure 4B). Kaplan-Meier survival analysis showed that the OS of patients in the high-risk group was significantly worse than that in the low-risk group (*P* < 0.001, Figure 4C). The distribution risk scores, duration of recurrence-free survival (RFS), and lncRNA expression levels were illustrated in Figure 4D. The number of deaths in the high-risk group was significantly higher than that in the low-risk group. These results suggest that the immune-related 56-lncRNA signature can distinguish high-risk patients from low-risk individuals, and it is of great significance in predicting the prognosis of BC.

### The LncRNA Signature as an Independent Prognostic Predictor

**FIGURE 5:**
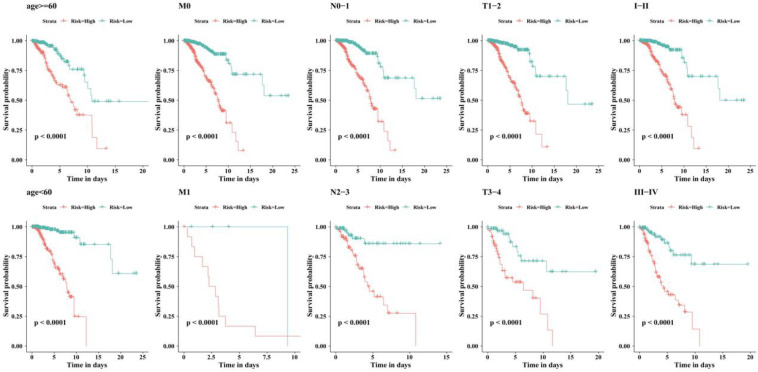
Kaplan-Meier survival curves of BC patients in different clinical subgroups.

We examined the independent prognostic ability of the immune-related lncRNA signature. The BC patients from the TCGA cohort were reassigned according to different prognostic and clinicopathological factors consisting of age, metastasis, and tumor staging (TNM and clinical staging). Kaplan-Meier survival analysis was performed for each subgroup and showed that the survival time of BC patients in the low-risk group was significantly prolonged regardless of age, TNM staging, and clinical staging (*p* < 0.0001, Figure 5). This indicates that the immune-related 56-lncRNA signature is independent of gender, age, and metastasis in survival prediction, which can be considered to be an independent predictor of the prognosis of BC patients.

### Validation of the LncRNA Signature and the Comparison Between the Signature and Known Prognostic Biomarkers

**FIGURE 6:**
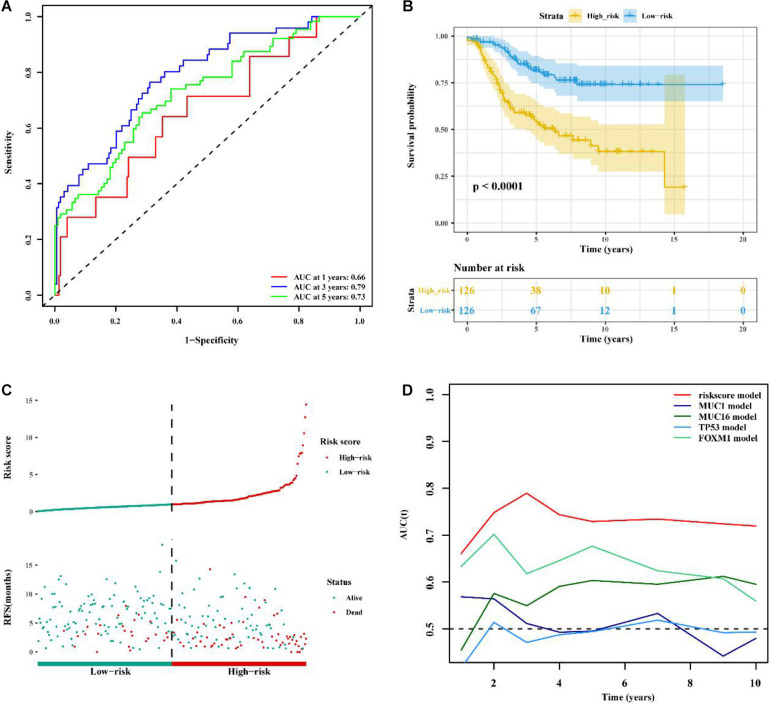
Comparison of prognostic performances between the lncRNA signature and known prognostic biomarkers and validation using the GSE21653 cohort. **(A)** The ROC curve to verify the prediction efficiency of the lncRNA signature. **(B)** Kaplan-Meier curve for the OS of BC patients in the high- and low-risk groups. **(C)** The risk-score distribution and duration of RFS of the lncRNA signature in the GSE21653 validation cohort. **(D)** Time-dependent ROC curve to compare prediction efficiency between the lncRNA signature and the known prognostic biomarkers.

**FIGURE 7:**
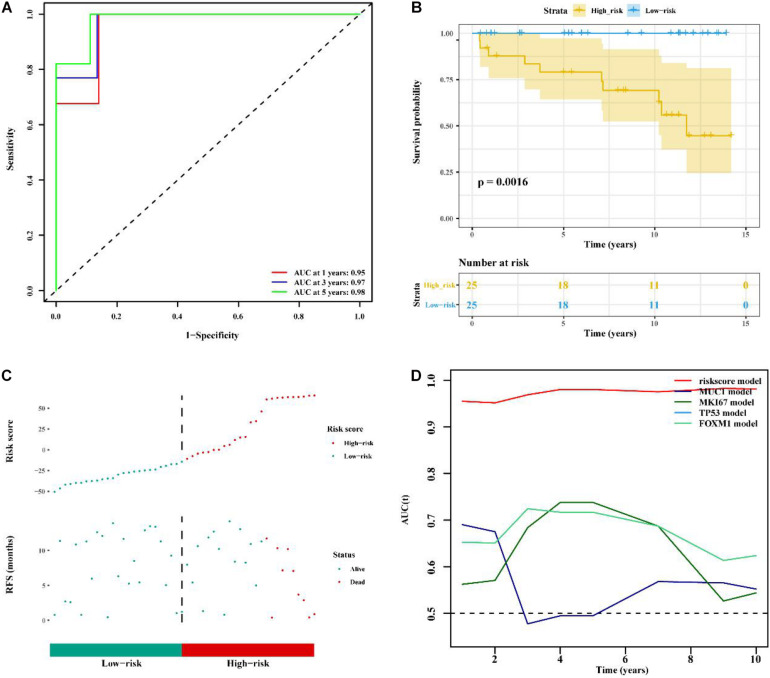
Comparison of prognostic performances between the lncRNA signature and known prognostic biomarkers and validation using the BC-KR cohort. **(A)** The ROC curve to verify the prediction efficiency of the lncRNA signature. **(B)** Kaplan-Meier curve for the OS of BC patients in the high- and low-risk groups. **(C)** The risk-score distribution and duration of RFS of the lncRNA signature in the BC-KR validation cohort. **(D)** Time-dependent ROC curve to compare prediction efficiency between the lncRNA signatures and known prognostic biomarkers.

To determine whether the immune-related 56-lncRNA signature was superior to known prognostic biomarkers in terms of prognostic capability and prediction efficiency, the validation was performed in the other two independent verification set GSE21653 data set and the BC-KR data set cohort. The ROC analysis showed a similar prediction efficiency between the training cohort and the GSE21653 validation cohort (Figure 6A). Kaplan-Meier survival analysis showed that the OS of BC patients in the high-risk group was significantly worse than that in the low-risk group (*P* < 0.001, Figure 6B). The distribution of risk scores and duration of RFS were illustrated in Figure 6C. The number of deaths in the high-risk group was significantly higher than that in the low-risk group. ROC analysis showed that the AUC of the lncRNA signature was higher than that of known prognostic biomarkers (Figure 6D). The lncRNA signature had better stability and reliability in predicting the survival of BC patients, which should be considered as a good prognostic biomarker. The validation using the BC-KR cohort showed similar results in prognostic capability and prediction efficiency of the signature, more deaths in the high-risk group, and the superiority over known biomarkers (Figure 7). These results suggest that the immune-related 56-lncRNA signature can predict the survival of BC patients in other independent cohorts.

### Univariate and Multivariate Cox Regression Analysis of Clinicopathological Factors Related to the BC Prognosis

**FIGURE 8:**
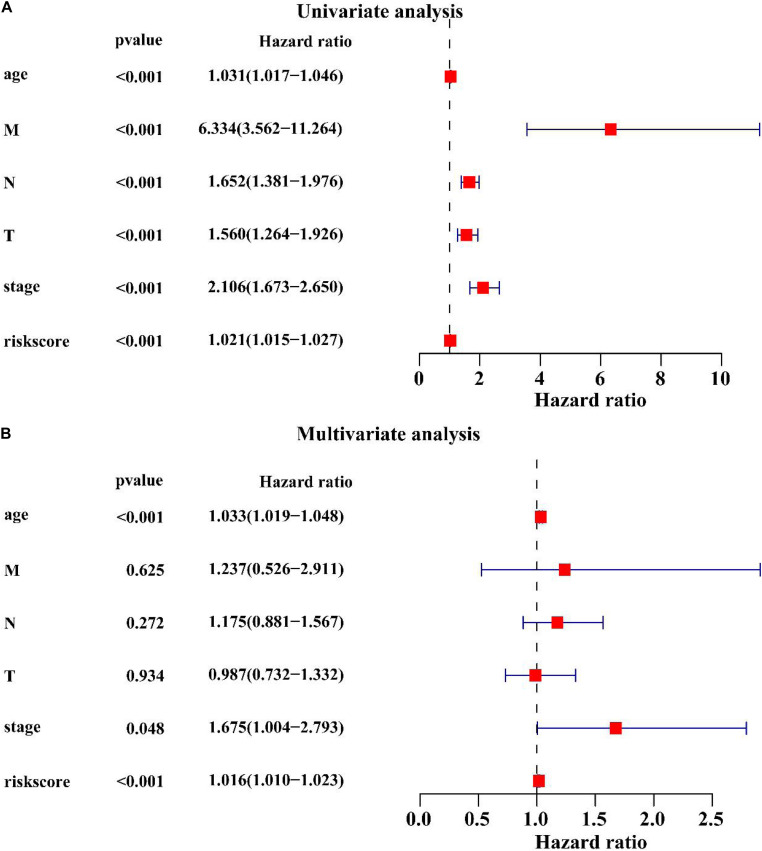
Univariate and multivariate Cox regression analysis of clinicopathological factors related to the BC prognosis. **(A)** The univariate forest plot illustrated the clinicopathological factors related to the BC prognosis. **(B)** The multivariate forest plot exhibited the clinicopathological factors related to the BC prognosis.

Univariate Cox regression analysis showed that age (HR = 1.031, *P* < 0.001), M stage (HR = 6.334, *P* < 0.001), N stage (HR = 1.652, *P* < 0.001), T stage (HR = 1.560, *P* < 0.001), clinical stage (HR = 2.106, *P* < 0.001), the lncRNA signature (HR = 1.021, *P* < 0.001) were associated with the BC prognosis (Figure 8A). Multivariate Cox regression analysis revealed that age (HR = 1.033, *P* < 0.001), clinical stage (HR = 1.675, *P* < 0.001) and the lncRNA signature (HR = 1.016, *P* < 0.001) were significantly associated with the BC prognosis (Figure 8B). These results suggest that the lncRNA signature is an important prognostic factor for BC.

### Construction of a Nomogram to Predict the OS of Patients With BC

**FIGURE 9:**
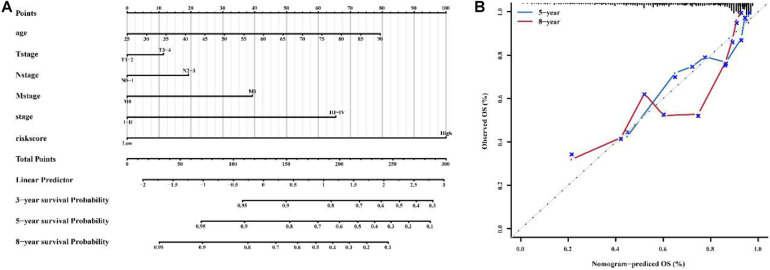
Construction of a nomogram to predict the OS of patients with BC. **(A)** nomogram to predict survival probability at 1, 3, 5 and 8 years for BC. **(B)** Calibration curve with Hosmer-Lemeshow test of the nomogram.

Combined with clinicopathological characteristics, we constructed a nomogram containing the immune-related 56-lncRNA signature (Figure 9) to predict the 3-, 5-, and 8-year survival rate of BC patients. The results suggest that the nomogram can independently evaluate the survival of BC patients, which may help physicians make better medical decisions and follow-up plans.

### GSEA and GSVA Analysis for Phenotype Differences Between the High- and Low-Risk Groups

**FIGURE 10:**
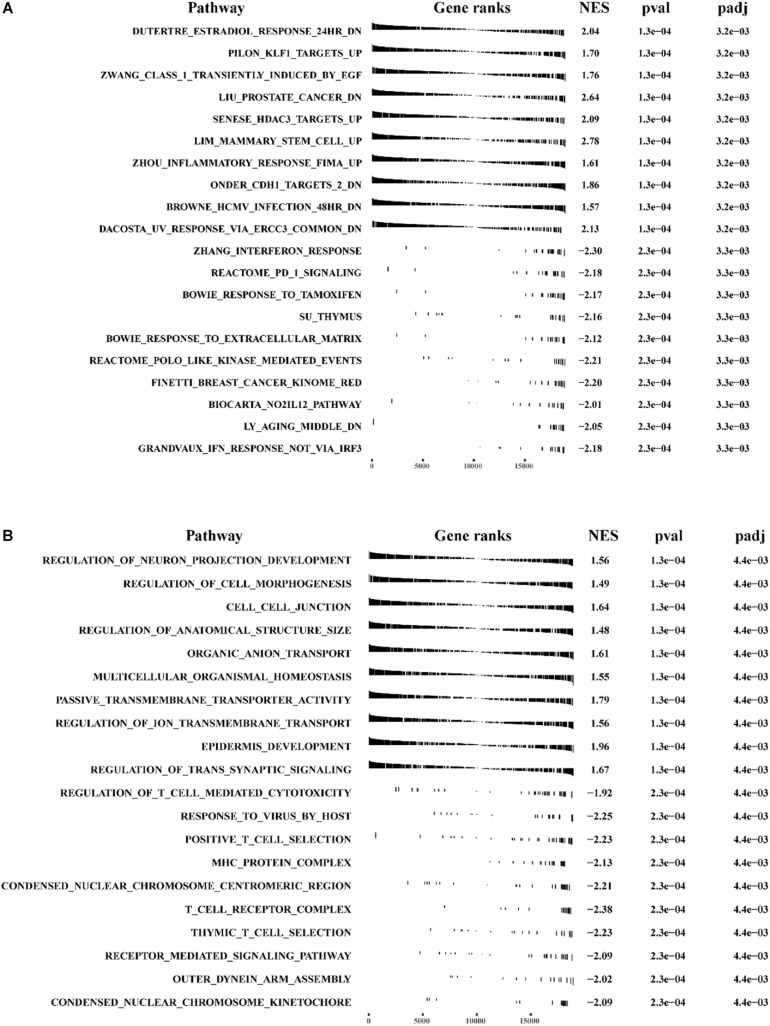
The GSEA analysis for pathway enrichment between the high-risk group and the low-risk group. GSEA enrichment analysis was performed using **(A)** “c2.all.v7.0 symbols.gmt” and **(B)** “c5.all.v7.0 symbols.gmt” as the reference gene sets.

We conducted GSEA and GSVA analysis to identify differences in main functional phenotypes between the high- and low-risk groups. As shown in Figures 10A,B and Supplementary Figure 2A, GSEA analysis revealed that the pathways involved in BC patients from the high-risk group consisted of CELL_CELL_JUNCTION, DUTERTRE_ESTRADIOL_RESPONSE_24HR_DN, EPITHEL IAL _MESENCHYMAL_TRANSITION, KRAS_SIGNALING _Up, Zhang_INTERFERON_ RESPONSE, and POSITIVE _T_CELL_SELECTION. GSVA analysis showed that HEME _ METABOLISM, TGF_BETA_SIGNALING, KRAS_SIGN ALING_UP, IL6_JAK_STAT3_ SIGNALING, INFLAMMA TORY_RESPONSE were activated in BC patients from the high-risk group, and ESTROGEN_RESPONSE_LATE, MYC_TARGETS_V1, DNA_REPAIR, and INTERFE RON_GAMMA_RESPONSE were inhibited (Supplementary Figure 2B and Supplementary Table 6).

### Correlation Between Immune Cell Infiltration and the LncRNA Signature

**FIGURE 11:**
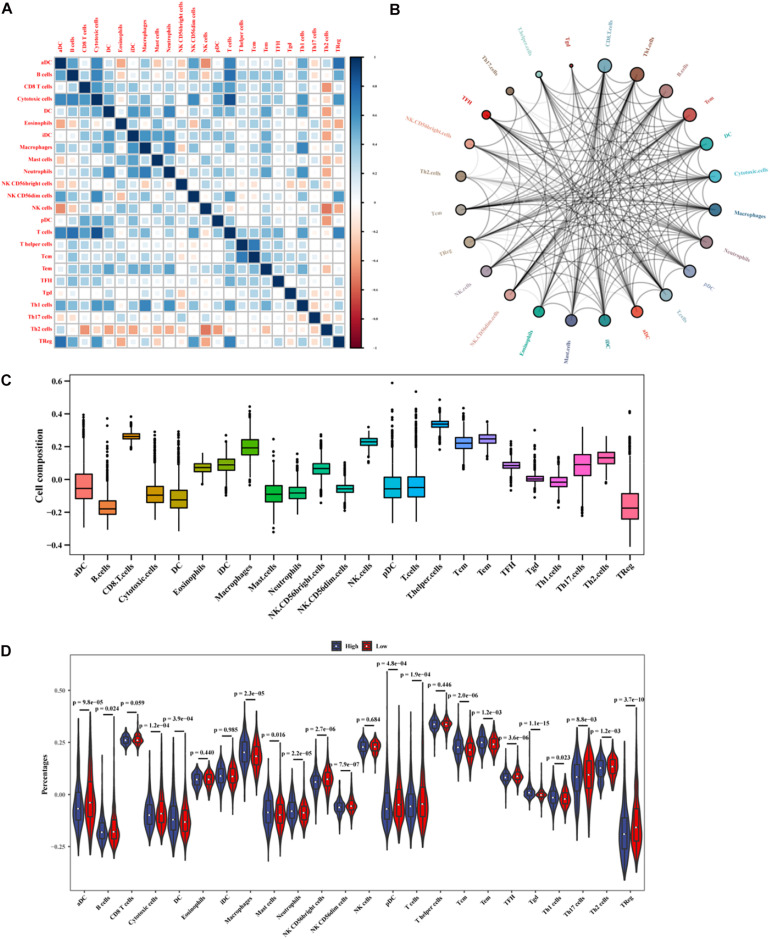
Analysis of immune cell infiltration in BC patients. **(A)** The correlation between infiltrating immune cells. **(B)** The cycle indicates the immune cell-cell interactions in the tumor microenvironment in BC. A larger circle corresponds to more interactions between immune cells and vice versa. **(C)** The *x*-axis represents immune cell types, and the *y*-axis represents the percentage of immune cells. **(D)** The differences in the expressions of infiltrating immune cells between the high- and low-risk groups.

**FIGURE 12:**
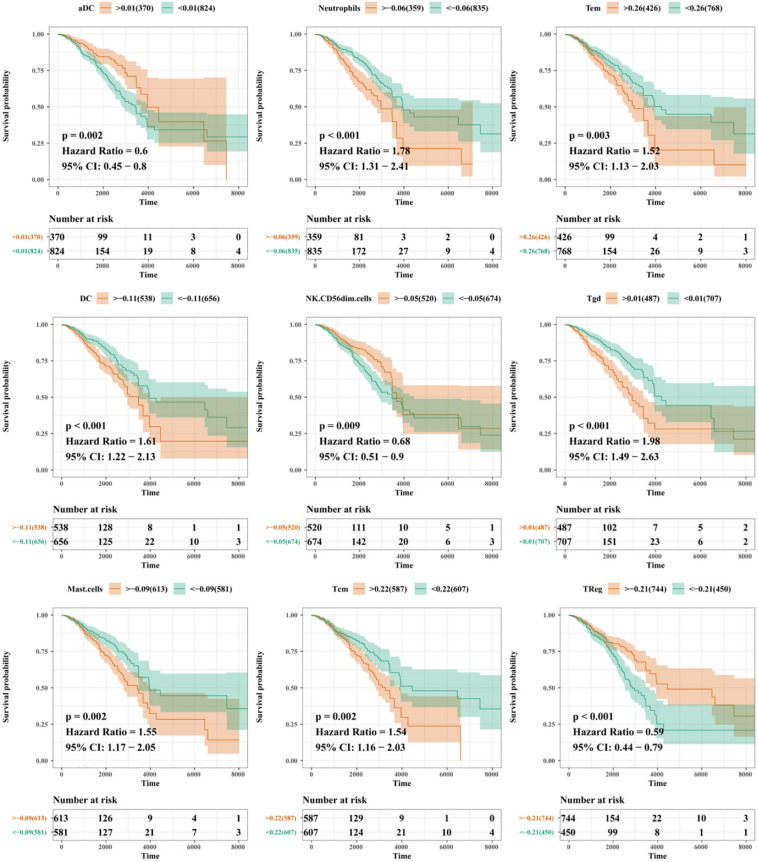
Prognosis analysis for immune cells.

Based on the mRNA-lncRNA co-expression network, GO enrichment analysis had confirmed that the immune-related lncRNA signature was involved in the occurrence and development of BC by regulating immune responses. To explore the association between the lncRNA signature and immune cell infiltration, a comprehensive analysis was carried out to figure out the composition of infiltrating immune cells in the tumor microenvironment. Correlation analysis showed that cytotoxic cells were positively correlated with T cells and B cells, while Th2 cells were negatively correlated with other immune cells (Figure 11A). We predicted the interaction network between immune cells, and the results showed that CD8^+^ T cells, Th1 cells, and B cells had a strong interaction with other immune cells, while Tgd, T helper cells, and Th17 cells had a weak interaction with other immune cells (Figure 11B). The analysis of immune cell composition showed that BC patients had higher numbers of T helper cells, CD8^+^ T cells, and T helper cells but lower numbers of B cells and regulatory T (TReg) cells in the tumor microenvironment (Figure 11C). We compared the difference in the expressions of infiltrating immune cells in BC patients between the high-risk group and the low-risk group. As shown in Figure 11D, the levels of aDC, Macrophages, Tgd, Macrophages, and TReg were significantly different between the two groups (*P* < 0.01). The prognosis analysis for infiltrating immune cells showed that aDC, Neutrophils, Tem, DC, NK CD56dim cells, Tgd, Mast cells, TCM, and TReg were correlated with the prognosis of BC patients (Figure 12). The correlation analysis confirmed a significant correlation between the infiltrating immune cells and the lncRNAs in the signature (Supplementary Figure 3). These results suggest that the lncRNAs in the signature have a complex regulatory relationship with immune cell infiltration, which may be an important way to participate in the development of BC and have high research value.

## Discussion

Breast cancer remains one of the most lethal malignancies in the world. Although currently available cancer treatments (such as surgery, chemotherapy, radiotherapy, endocrine therapy, targeted therapy, and immunotherapy) have a positive impact on BC survival, increasing life expectancy in a short period seems unrealistic. The risk of BC recurrence is particularly high, suggesting that BC recurrence is one of the most important causes of mortality. The difficulty of predicting the BC prognosis among a complex tumor microenvironment comprised of molecular and cellular heterogeneity between some commonly used markers in several cancers, such as CA 15-3, CA 27.29, CEA, MUC1, MUC16, TP53, FOXM1, ER, PR, HER2, KI 67, and others (Børresen-Dale, 2003; Lakshmanan et al., 2012; Graham et al., 2014; Kabel, 2017; Song et al., 2017; Stergiou et al., 2019), makes a prediction inaccurate. Therefore, the proposal of an effective and reliable biomarker using a risk prediction model and cohort-specific imputation of gene expression to encourage the implementation of relevant clinical trials to validate this biomarker is currently possible. Advantages of a reliable biomarker are that high-risk populations can be closely monitored, management for the prevention and early detection of BC recurrence can be implemented, and once the recurrence is timely detected, individualized treatment plans for prolonged survival can be prescribed.

Previous studies of prognostic biomarkers of BC have focused on coding genes and microRNAs. LncRNAs, especially immune-related lncRNAs, recently emerging as novel biomarkers have gained primary results. In-depth sequencing studies have found that lncRNAs directly interact with a variety of signaling pathways and regulators of oncogenes or tumor suppressor genes, thereby affecting tumor occurrence and development (Lin and Yang, 2018).

In this study, we initially identified 948 immune-related lncRNA candidates. Using a signature risk prediction model and the iterative Lasso Cox regression analysis, 56 lncRNAs with the maximum prognostic values were selected from the candidates to form a signature. ROC analysis showed good prognostic efficiency, with an AUC of 0.86. Finally, a 56-lncRNA signature was constructed. In the test set, BC patients were divided into the high- and low-risk groups according to their risk scores. Kaplan-Meier curve showed that patients in the low-risk group had a significantly longer OS than those in the high-risk group. Moreover, multivariate Cox regression analysis showed that the lncRNA signature was an independent predictor for BC prognosis in the two other validation cohorts from GEO and ICGC. Besides, the lncRNA signature offers a more effective prediction than known biomarkers. Clinical subgroup analyses showed the signature was independent of the presence of clinicopathological factors (gender, age, metastatic lymph nodes, and tumor metastasis) related to BC prognosis, indicating that the signature is an independent predictor of BC survival and the perspective of its clinical applicability is expected. The construction of a nomogram confirmed that the lncRNA signature can independently evaluate BC survival.

Cancer immunotherapy is a rapidly developing and exciting field of oncology. Eliciting adaptive immunity, particularly the adaptive infiltrating immune system which is an important component in the tumor microenvironment, is the goal of immunotherapy for effective and sustained anti-tumor responses to limit tumor growth and metastasis. Studies have shown that a combination of immunotherapy and other therapies can bring better benefits to patients with BC, especially advanced-stage, triple-negative BC (Schmid et al., 2018). In this study, GEVA analysis of mRNAs that are co-expressed with immune-related lncRNAs showed that TGF_BETA_SIGNALING and IL-6/JAK/STAT3TGF were the main enriched pathways. Hao et al. (2017) reported that TGF - β signaling pathway plays an important role in regulating the migration and invasion of lung cancer and BC cells. The IL-6/JAK/STAT3 signaling pathway is critical in regulating cell growth, survival, and differentiation in tumor cells, and also in mediating the differentiation and activation of T lymphocytes (Eskiler et al., 2019). Immune-related lncRNAs may participate in the occurrence and development of BC via directly interacting with a variety of signaling pathways and regulators of oncogenes or tumor suppressor genes to regulate adaptive immune responses. We further studied the relationship between lncRNAs in the signature and immune cell infiltration, and found that the levels of aDC, B cells, NK, CD56dim cells, T cells, TFH, and TReg in the low-risk group were higher than those in the high-risk group, ADC, NK, CD56DIM cells and TReg were correlated with BC prognosis, and ADC, NK, CD56DIM cells were positively correlated with T cells. Tumor-infiltrating lymphocytes are an important component of the tumor microenvironment in BC. Pantelidou et al. (2019) reported that Olaparib, a PARP inhibitor, inhibited tumor via activating the cGAs/STING pathway in tumor cells to induce T cell recruitment.

Pei et al. (2018) reported that LNC SNHG1 functioning as a competing endogenous RNA (CeRNA) inhibited the differentiation of TReg cells, thereby preventing BC immune escape. Wang et al. (2019) reported that TReg cells play a major role in the development of immunosuppressive tumor microenvironment mainly through cytokine signal transductions–a key determinant of immunosuppressive potential and unfavorable clinical outcomes. Inconsistent with these findings, we found that BC patients with a high TReg level had a better prognosis than those with a low TReg level (Takasato et al., 2020). The origin of tumor-infiltrating TReg cells and their association with circulating TReg cells are rarely reported, which will be explained in the follow-up study in our following publications.

Besides, there are some limitations of our study. Further cell and animal experiments are needed to verify the correlation between lncRNA expressions and immunophenotype in BC. To explore the underlying immune mechanisms, a larger data set is needed to verify the accuracy of the prediction model.

## Conclusion

In conclusion, we have constructed an immune-related 56-lncRNA signature which can be used as an independent prognostic marker for the survival risk subgroup of BC patients. This lncRNA signature is superior to known predictors in terms of prognostic performance in risk stratification, which will renew hope for experimental and clinical validations as well as clinical application in the future.

## Data Availability Statement

The original contributions presented in the study are included in the article/Supplementary Material, further inquiries can be directed to the corresponding author/s.

## Author Contributions

CY, WH, and NL conceived and designed the study. ZH, CX, and LY performed the experiments. FZ, WH, and ZZ analyzed the data. ZH wrote the manuscript. All authors have read and approved this manuscript.

## Conflict of Interest

The authors declare that the research was conducted in the absence of any commercial or financial relationships that could be construed as a potential conflict of interest.

## Footnotes

1 https://xenabrowser.net/datapages/

2 https://dcc.icgc.org/

3 https://ImmPort.niaid.nih.gov
