# Supplementary figures and images for: A Novel Framework to Predict Breast Cancer Prognosis Using Immune-Associated LncRNAs
Source: Front Genet. 2021 Jan 21;11:634195. doi: 10.3389/fgene.2020.634195 (PMC7873981; doi:10.3389/fgene.2020.634195)

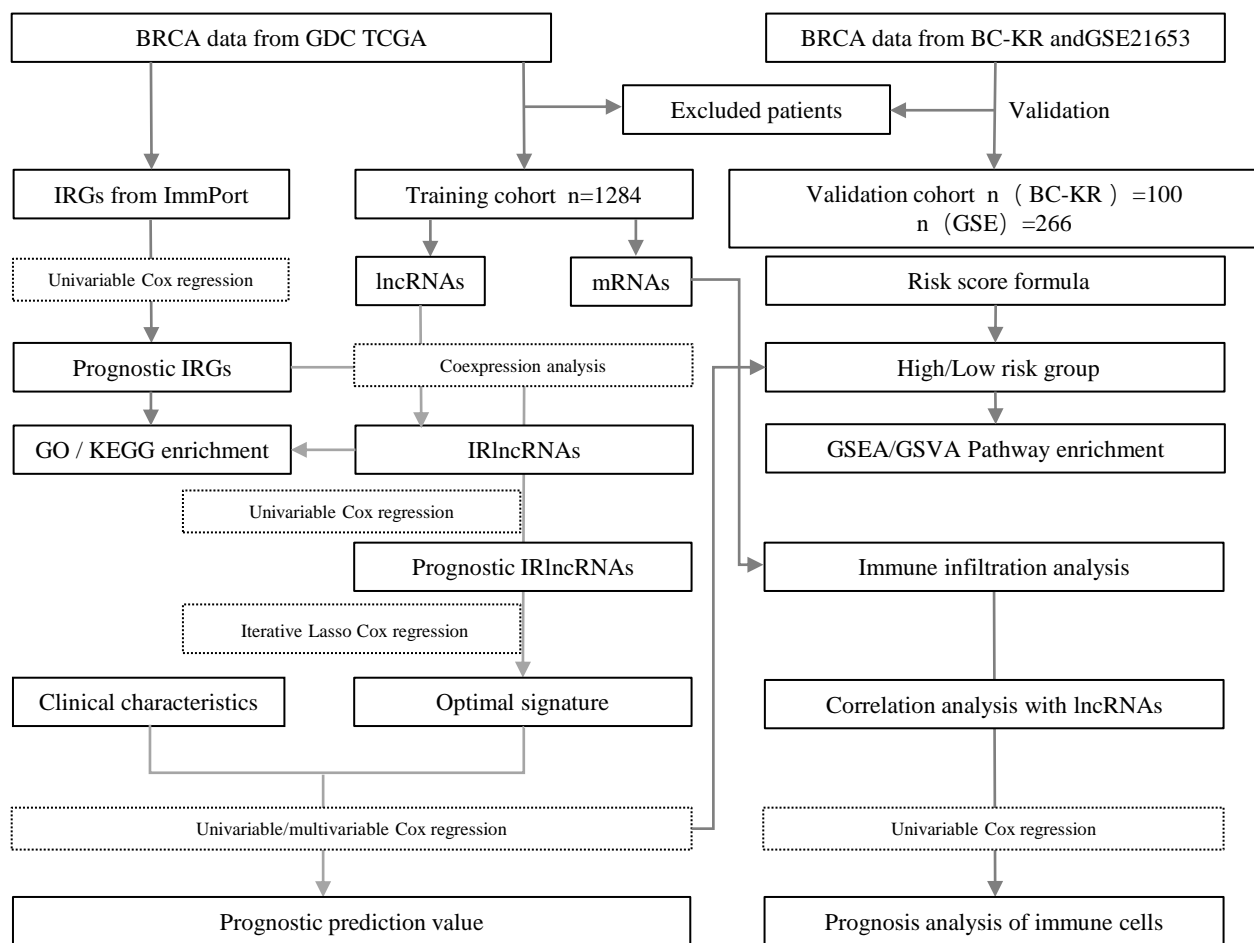

Figure S1. Flowchart for bioinformatics analysis of publicly available data.

Supplement: Supplementary file 1 [file Data_Sheet_1.PDF]
