# Supplementary material for: A Novel Framework to Predict Breast Cancer Prognosis Using Immune-Associated LncRNAs
Source: Front Genet. 2021 Jan 21;11:634195. doi: 10.3389/fgene.2020.634195 (PMC7873981; doi:10.3389/fgene.2020.634195)
Supplement: Supplementary file 2 [file Data_Sheet_2.PDF]

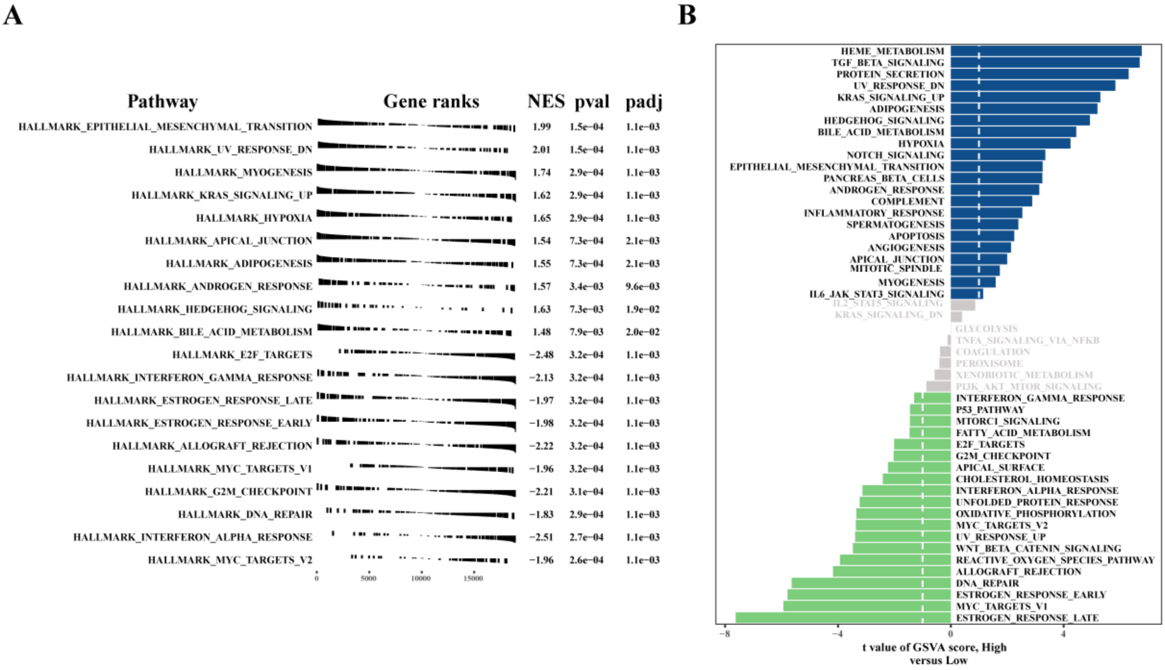

Figure S2. GSEA analysis for pathway enrichment between the high-risk group and the low-risk group. GSEA enrichment analysis using (A) “h.all.v7.0 symbols.gmt” and (B) “h.all.v7.0 symbols.gmt” as the reference gene sets.
