# Supplementary material for: A Novel Framework to Predict Breast Cancer Prognosis Using Immune-Associated LncRNAs
Source: Front Genet. 2021 Jan 21;11:634195. doi: 10.3389/fgene.2020.634195 (PMC7873981; doi:10.3389/fgene.2020.634195)
Supplement: Supplementary file 3 [file Data_Sheet_3.PDF]

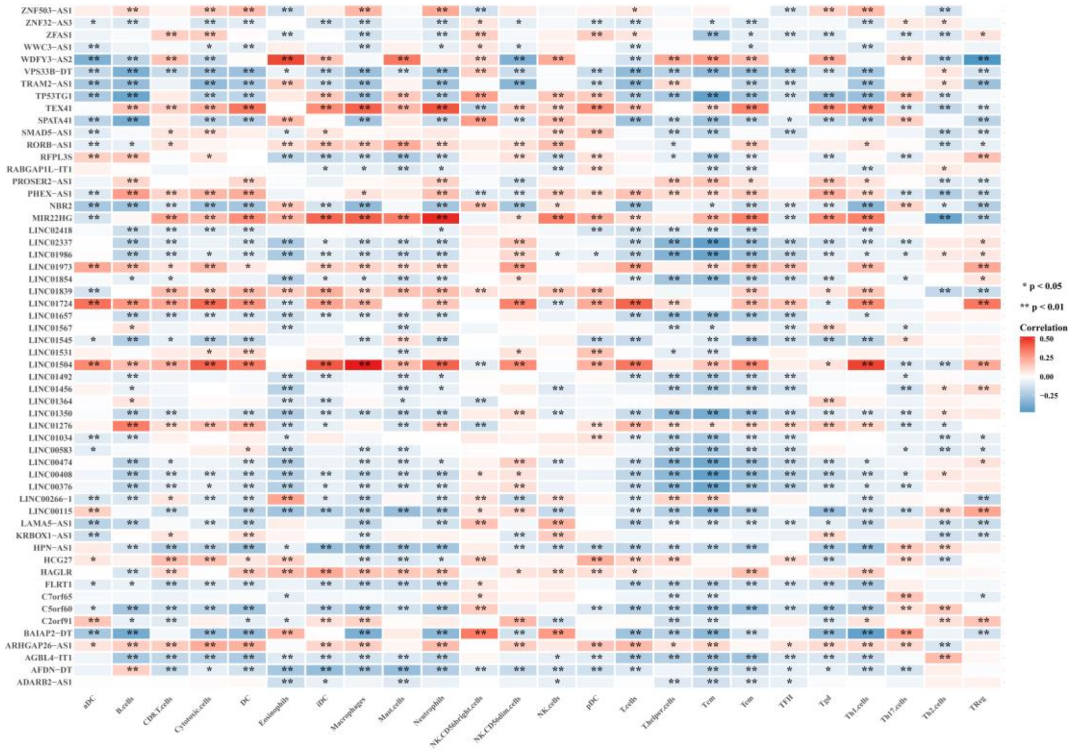

Figure S3. Correlation analysis between lncRNAs in the signature and infiltrating immune cells. The x-axis represents immune cell types, and the y-axis represents 56 lncRNAs. Red indicates positive correlations and blue indicates negative correlations. The intensity of the color corresponds to the correlation coefficient: the stronger the correlation, the darker the color.
